# A Survey on Chemical Constituents and Indications of Aromatic Waters Soft Drinks (Hydrosols) Used in Persian Nutrition Culture and Folk Medicine for Neurological Disorders and Mental Health

**DOI:** 10.1177/2156587217714145

**Published:** 2017-06-21

**Authors:** Azadeh Hamedi, Ardalan Pasdaran, Zahra Zebarjad, Mahmoodreza Moein

**Affiliations:** 1 Medicinal Plants Processing Research Center, Shiraz University of Medical Sciences, Shiraz, Iran

**Keywords:** essential oil, neurological disorders, hydrosol, Aragh, aromatic waters, distillate

## Abstract

In Persian nutrition culture, drinking aromatic waters (hydrosols, distillate) has a long history as functional beverages or therapeutic remedies. The co-distilled water with essential oils, which contains partial amounts of more water-soluble volatile compounds are diluted and used as beverages. Since the solubility of volatile components is different in water, the overall composition, and thus the biological activities of aromatic waters seem to be different from the essential oils they were co-distilled with. Despite the essential oils, chemical constituents of many aromatic waters have not been evaluated scientifically. This research investigated hydrosols used for mental and neurological health maintenance in Persian nutrition culture and their chemical constituents. Constitutions of these hydrosols were extracted by liquid/liquid extraction method and identified by gas chromatography–mass spectrometry. Furthermore, cluster analysis was used to evaluate the relevance of these hydrosols chemical constituents. About 93 compounds were identified from 20 aromatic waters. the major or second major constituents were thymol (azarol howthorn, frankincense, lemon balm, valerian, shadab), phenethyl alcohol (damask rose, dog-rose, starflower), carvacrol (basil, creeping buttercup, lemon balm); eugenol (shadab, dog-rose, starflower, basil), camphor (yarrow and wormwood), carvone (oriental plane), caryophyllene (cuminum), cinnamaldehyde (Chinese cinnamon), *p*-cymen-7-ol (musk willow), limonene (lemon verbena), linalool and α-terpineol (bitter orange), menthol (date palm) and methyl 5-vinylnicotinate (olive). Although, these hydrosols prepared from plants belong to different genus and families, but cluster analysis showed obvious similarities between their chemical constituents. Results of this investigation showed in many cases that the constituents of aromatic waters are different from the pure essential oil.

Mental disorders are one of the most depilating diseases that in compression to other chorionic conditions have a higher prevalence in different societies.^1^ They have been clearly documented for accompanying to the many serious chronic illnesses.^2^ A diverse range of neurological disorders symptoms, including anxiety, depression, phobia, tension, headache, insomnia, and others have a great impact on patient quality of life as well as dynamics and health status of communities.^3^ These neurological disorders affect a large number of populations, for example, major depression, based on the World Health Organization reports, is the fourth cause of disability disorders, which affects 121 million people worldwide.^4^

For centuries, traditional herbal formulations and different functional foods have been widely used for treatment of various mental and neurological conditions. In Persian traditional medicine many aromatic plants and their derivatives such as hydrosol have been used as functional beverages for mental and neurological disorders. In Persian traditional medicine system, therapeutic remedies divided by the nature of drugs origins. Based on this classification the remedies could have hot, cold, dry, wet, or moderate nature.^5^ In Persian traditional medicine systems, several hydrosol drinks obtained from different medicinal plants have been used for a range of neurological conditions. Different therapeutic effects have been cited for them such as antianxiety, sedative, anticonvulsant, antifatigue, and analgesics for headaches.

Pervious investigations on biological activity of medicinal plants on neural system showed diverse mechanisms of action, including upregulating of monoamine neurotransmitters by suppressing the reuptake, inhibiting monoamine oxidases, simulating of brain-derived neurotrophic factor expression, blocking 5-HT1A receptor and promoting the secretion of adrenocorticotropin for some of them.^6,7^

Although extensive evidences showed potential effects of phytochemicals on neurological disorders but a few researches focused on volatile constituents of traditional formulation such as hydrosols or aromatic waters.^8^ Aromatic water beverages constitute the major part of herbal market in Iran, more than 50 different types of these products present as functional drinks. The diverse origin of these products caused a very diverse volatile constituents and therapeutic activity. Although in some cases, these aromatic waters have a similar aroma to the pure essential oils they were co-distilled with, but in many cases, they have different volatile constituents due to different water solubility of the volatile compounds and thus these have different properties.

This study investigated constituents of aromatic waters used in Persian nutrition culture and folk medicine for neurological conditions.

## Materials and Methods

### Phytochemical Analysis

Names and therapeutic properties of aromatic waters used for different neurological disorders were obtained using questioners filled by manufactures and retail sellers of these aromatic waters in Fars province (2016-2017). The most frequently cited aromatic waters were purchased from the herbal market and their constituents were investigated. Briefly, 500 mL of each aromatic water was extracted with 500 mL of petroleum-ether. The essential oils of the samples were extracted from aqueous phase to organic phase (petroleum-ether) using a glass liquid-liquid extractor. In order to increase the concentration of volatile component in the organic phase, the aqueous phase was replaced by the fresh hydrosol after 150 minutes. Petroleum-ether extract was concentrated by rotary evaporator (IKA RV10), equipped with a Heidolph Rotavac vacuum pump.

### Gas Chromatography–Mass Spectrometry

The concentrated petroleum-ether extracts of the beverages were subjected to gas chromatography–mass spectrometry (Agilent Technologies 7890 Gas Chromatograph) for analysis of the chemical compositions equipped with HP-5MS capillary column (Agilent Technologies 19091 S-433., 30 × 0.25 mm inner diameter). Mass detector was Agilent Technologies model 5975 C in EI mode at 70 eV. The thermal ramp rates were increasing temperature from 60°C to 220°C with the rate of 5°C/min and held at 220°C for 10 minutes. The carrier gas (helium) was used with the flow rate of 1 mL/min. The interface temperature and mass range was set up to 280°C and 30 to 600 *m*/*z*, respectively. Identification of the volatile compounds was done using the NIST (National Institute of Standards and Technology) or Wiley libraries, pervious literature, and by comparison with retention times and mass spectra of the reference compounds.^9,10^

### Statistical Analysis

In order to find correlation between aromatic waters constituents, hierarchical cluster analysis and K-means analysis were done using SPSS statistics software package (version 16).

## Results and Discussion

The beverages that are used for neurological disorders in Persian traditional medicine are listed in Table 1. Some of these beverages and their applications have been maintained in traditional Persian manuscripts such as *Qarabadin-e-kabir* and *Qarabadin-e-salehi* and some others recently have become popular in folk medicine without any citation in traditional literatures. The hydrosols that are used in this study prepared from plants belong to 15 families (Table 1). Most of these hydrosol beverages are prepared from the leaves and flowers of plants. Diverse effects on neurological conditions have been cited for these aromatic waters, including memory improvement, antidementia, sedative, analgesic, antiepileptic, neurological pain killer, antidepressant, antihysteria, and antianxiety. From the point of view of Iranian folk medicine, most of these beverages have hot nature (Table 2). Sedative effect was the most frequent therapeutic application of these hydrosol beverages.

**Table 1.** Plants Name and Their Medicinal Parts That Are Used to Prepare Aromatic Waters for Neurological Disorders or Maintaining Mental Health.

Aromatic Waters Beverage Name	Aromatic Water Name in Persian	Scientific Name	Family	Plant Parts
Azarol hawthorn	Aragh-e-Keyalak	*Crataegus azarolus* var. *chlorocarpa* (Moris) K.I.Chr.	Rosaceae	Leaf and fruits
Basil	Aragh-e-Reyhan	*Ocimum basilicum* L	Lamiaceae	Aerial parts
Bitter orange	Aragh-e- Bahar Naranj	*Citrus aurantium*	Rutaceae	Flowers
Chinese cinnamon	Aragh-e-Darchin	*Cinnamomum cassia* (L.) J.Presl	Lauraceae	Stem bark
Creeping buttercup	Aragh-e-Alaleh	*Ranunculus repens* L	Ranunculaceae	Flowers
Cunninum	Aragh-e-Ziereh	*Cuminum cyminum* L	Apiaceae	Seed
Date palm	Aragh-e-Tarooneh	*Phoenix dactylifera* L	Arecaceae	Spathe
Damask rose	Golab	*Rosa × damascene*	Rosaceae	Flowers
Dog-rose	Aragh-e-Nastaran	*Rosa canina*	Rosaceae	Flowers
Frankincense	Aragh-e-Kondor	*Boswellia* sp	Burseraceae	Ole-gum-resin
Lemon balm	Aragh-e-Badranjbooye	*Melissa officinalis* L	Lamiaceae	Leaf
Lemon verbena	Aragh-e-Beh Limoo	*Aloysia citriodora Palau*	Verbenaceae	Leaf
Musk willow	Aragh-e-Bidmeshk	*Salix aegyptiaca* L	Salicaceae	Catkins
Olive	Aragh-e-Zeytoon	*Olea europaea* L	Oleaceae	Leaf
Oriental plane	Aragh-e- Chenar	*Platanus orientalis* L	Platanaceae	Leaf
Starflower	Aragh-e- Gol Gavzaban	*Echium amoenum* Fisch & C.A.Mey	Boraginaceae	Flowers
Valerian	Aragh-e-Sonbol tib	*Valeriana officinalis* L	Caprifoliaceae	Aerial parts
Wormwood	Aragh-e-Dermaneh	*Artemisia sieberi* Besser	Asteraceae	Aerial parts
Yarrow	Aragh-e-boomadaran	*Achillea millefolium* L	Asteraceae	Aerial parts
A polyherbal hydrosol	Aragh-e-Shadab	A mixture of *Ocimum basilicum* L, *Aloysia citriodora* Palau, *Echium amoenum* Fisch & C.A.Mey, *Salix aegyptiaca* L, *Valeriana officinalis* L, *Cinnamomum cassia* (L.) J.Presl, *Ranunculus repens* L *Tanacetum parthenium* (L.) Sch.Bip.		

**Table 2.** Aromatic Waters Indications in Mental Health Conditions as Well Mental Disorders Treatment.

Aromatic Waters Beverage Name	Nature	Indications	Dosing
*Monoherbal aromatic waters*			
Azarol hawthorn	Cold nature	Anticonvulsant	100 mL TID, before meal
Basil	Hot nature	Sedative, anti-hysteria	100 mL TID, after meal
Bitter orange	Hot nature	Neurotonic, antidizziness, antihysteria, sedative, antidepressant	100 mL TID, after meal
Cardamom	Hot nature	Neuralgic pain treatment, hypnotics, sedative, headache treatment	100 mL TID, after meal
Chinese cinnamon	Hot nature	Neurotonic, obsessive treatment, phobia treatment	100 mL TID, after meal
Clove	Hot nature	Antianxiety, neurotonic, headache treatment, anticonvulsant	100 mL TID, after meal
Common purslane	Cold nature	Headache treatment	100-150 mL ID, before meal
Common thyme	Hot nature	Anticonvulsant, neuralgic pain treatment	100 mL TID, before meal
Coriander	Cold nature	Obsessive treatment, antihysteria, brain improvement	100 mL QID, before meal
Costmary	Hot nature	treat unilateral headache, neuralgic pain treatment	100 mL TID, after meal
Cottonwood	Hot nature	Neurotonic, paralysis treatment, antitremor, numbness treatment	100 mL TID, after meal
Creeping buttercup	Cold nature	Analgesic for neuralgic pain, sedative, antihysteria	100 mL TID, after meal
Cuminum	Hot nature	Neurotonic	100 mL TID, after meal
Damask rose	Hot nature	Mental refreshing, sedative, brain improvement, antifatigue, neurotonic	100 mL TID, after meal
Dragonhead	Cold nature	Brain improvement, sedative, heart beating treatment, anticonvulsant, memory improvement, headache treatment	100 mL TID, after meal
Date palm	Hot nature	Neurotonic, sedative	150 mL TID, before meal and bedtime
Dog-rose	Hot nature	Sedative, neurotonic	100 mL TID, after meal
Felty germander	Cold nature	Tonic, anticonvulsant	100-150 mL TID, before meal
Frankincense	Cold nature	Dementia prevention, memory improvement, mindfulness	100 mL TID, after meal
Lavender	Hot nature	Hypnotics, sedative, headache prevention, anticonvulsant, antidizziness, antitremor	100 mL TID, after meal
Lemon verbena	Cold nature	Memory improvement, antidizziness, analgesic for neuralgic pain, sedative, antihysteria, treating unilateral headache pain	100 mL TID, after meal
Marjoram	Hot nature	Sedative, treat headache, anticonvulsant	100 mL TID, after meal
Musk willow	Cold nature	Sedative, anticonvulsant	100 mL TID, after meal
Persian hogweed	Hot nature	Hysteria treatment, anticonvulsant, memory improvement	100 mL TID, after meal
Olive leaves	Cold nature	Memory improvement, headache treatment, tooth pain treatment	100-150 mL TID, before meal
Oriental plane	Cold nature	Neurotonic	50-100 mL TID, after meal
Starflower	Cold nature	Neurotonic, sedative, obsessive treatment, antianxiety	100 mL TID, before meal
Valerian	Hot nature	Neurotonic, sedative, anticonvulsant, neurotonic, analgesic	100 mL QID, before meal and bedtime
Wormwood	Hot nature	Sedative, neurotonic, headache treatment, hypnotic	50-100 mL TID, after meal
Yarrow	Hot nature	Anticonvulsant, neurotonic	100 mL TID, after meal
Ziziphora	Hot nature	Sedative	100 mL TID, after meal
*Polyherbal aromatic waters*			
Shadab	Hot nature	Sedative, headache prevention, anticonvulsant, antidizziness	100 mL TID, before meal

Abbreviations: TID, 3 times a day; QID, 4 times a day.

**Figure 1.**
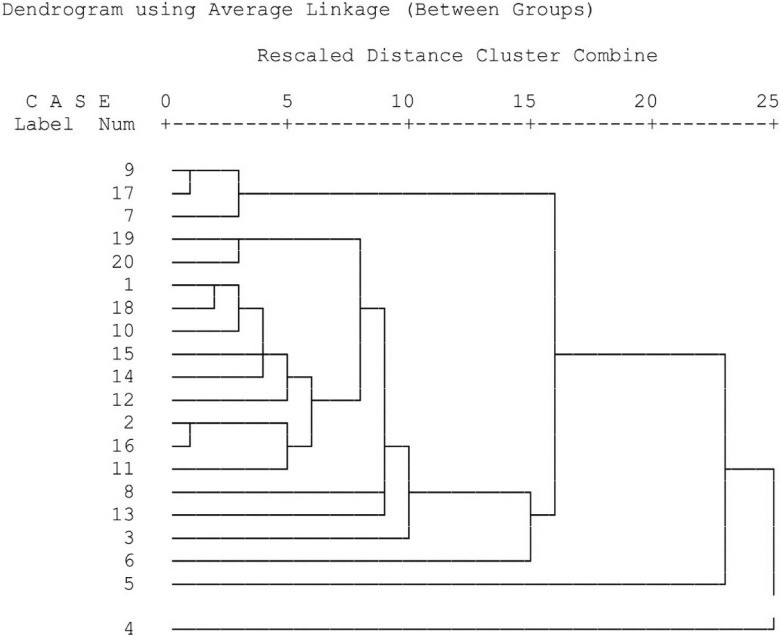
Cluster analysis of aromatic waters constituents (hierarchical cluster analysis). The aromatic waters are 1, azarol hawthorn; 2, basil; 3, bitter orange; 4, Chinese cinnamon; 5, creeping buttercup; 6, cuminum; 7, damask rose; 8, date palm; 9, dog-rose; 10, frankincense; 11, lemon balm; 12, lemon verbena; 13, musk willow; 14, olive; 15, oriental plane; 16, shadab; 17, starflower; 18, valerian; 19, wormwood; and 20, yarrow.

The chemical constituents of investigated aromatic waters were determined by gas chromatography–mass spectrometry technique and the identified compounds are listed in Table 3. Since the plants that are used to prepare these aromatic waters belong to different genus and families, hierarchical cluster analysis and K-means analysis based on chemical constitutes were used to make clusters and subclusters and find any correlations between aromatic waters and their constituents (Figure 1, Table 4). As seen in Table 3, which shows the constituents of beverages, the major or second major constituents were thymol (azarol howthorn, frankincense, lemon balm, valerian, shadab), phenethyl alcohol (damask rose, dog-rose, starflower), carvacrol (basil, creeping buttercup, lemon balm); eugenol (shadab, dog-rose, starflower, basil), camphor (yarrow and wormwood), carvone (oriental plane), caryophyllene (cuminum), cinnamaldehyde, (Chinese cinnamon), ρ-cymen-7-ol (musk willow), limonene (lemon verbena), linalool and α-terpineol (bitter orange), menthol (date palm), methyl-5-vinylnicotinate (olive), and yamogi alcohol (yarrow). There is a correlation between hierarchical cluster analysis and K-means analysis mean analysis results (Figure 1, Table 4). Based on both analyses damask rose, dog-rose, and starflower aromatic waters made a distinct cluster because of the presence of 47% to 77% phenethyl alcohol. The similarity of wormwood and yarrow aromatic waters seen in hierarchical cluster analysis and K-means cluster analysis might be because of the presence of camphor (23.56%-42.49%) as the major constituent, artemisia alcohol (3.12%-8.16%) and *trans*-thujone (4.36%-6.74%) in these aromatic waters. Considering hierarchical cluster analysis and K-means analysis, cuminum, creeping buttercup, and Chinese cinnamon constituents had a big difference with other aromatic waters.

**Table 3.** Aromatic Water Constituents Resulting From Gas Chromatography–Mass Spectrometry Analysis.

Component	Azarol hawthorn	Basil	Bitter orange	Chinese cinnamon	Creeping buttercup	Cuminum	Damask rose	Date palm	Dog—rose	Frankincense	Lemon Balm	Lemon verbena	Musk willow	Olive	Oriental plane	Shadab	Starflower	Valerian	Wormwood	Yarrow
1,8-Cineole	—	2.81	—	—	—	—	—	—	—	0.48	—	3.82	3.51	1.24	—	1.72	—	—	18.21	7.53
2,3-Dimethoxytoluene	—	—	—	—	—	—	—	—	—	—	—	—	—	—	1.83	—	—	—	—	—
3,4-Dimethoxytoluene	—	—	—	—	—	19.16	—	—	—	—	—	—	—	—	—	—	—	—	—	—
2,6-Dimethoxytoluene	—	—	—	—	—	2.54	—	—	—	—	—	—	—	—	—	—	—	—	—	—
Apiol	—	—	—	—	—	—	—	—	—	—	1.61	—	—	—	—	—	—	—	—	—
Anethol (*cis*)	—	—	—	—	—	—	—	—	—	—	1.23	—	—	—	—	—	—	—	—	—
Anethole (*trans*)	—	1.71	—	—	—	—	—	—	—	—	—	—	18.17	—	—	—	—	2.14	—	—
Artemisia alcohol	—	—	—	—	—	—	—	—	—	—	—	—	—	—	—	—	—	—	3.12	8.15
Ethylbenzene	—	—	—	—	—	—	—	—	—	—	—	—	—	—	2.03	—	—	—	—	—
Borneol	—	—	—	—	0.95	—	—	—	—	—	—	4	—	—	—	—	—	—	1.73	3.67
Benzeneacetonitrile	—	—	0.75	—	—	—	—	—	—	0	—	—	—	—	—	—	—	—	—	—
Camphor	—	2.66	—	—	—	—	—	—	—	0	—	—	—	—	—	1.8	—	—	23.56	42.49
Carvacrol	—	23.54	—	—	87.69	—	—	5.69	—	11.47	32.17	—	0.45	—	—	14.51	—	7.13	1.28	—
Carveol (*trans*)	—	—	—	—	—	—	—	—	—	2.28	—	0.77	—	—	—	—	—	—	—	—
Carvone	—	—	—	—	—	—	—	7.69	0	0.94	3.16	—	—	1.81	24.21	3.26	—	—	—	—
Caryophyllene (*trans*)	—	—	—	—	—	58.48	—	—	—	—	—	9.5	—	—	—	—	—	—	—	—
Caryophyllene oxide	—	—	—	—	0	11.84	—	—	—	—	—	0.72	—	—	—	—	—	—	—	—
Cinnamaldehyde, (*E*)	—	—	—	84.28	—	—	—	—	—	—	—	—	—	—	—	—	—	—	—	—
Cinnamaldehyde, (*Z*)	—	0	0	3.94	0	—	—	—	—	0	—	—	—	—	—	—	—	—	—	—
Citronellol	—	0	0	—	—	—	12.69	—	8.26	0	—	—	—	—	—	—	6.78	—	—	—
Chrysanthenone	—	—	—	—	—	—	—	—	—	2.52	—	—	—	—	—	—	—	—	—	—
Cumin aldehyde	—	—	—	—	—	—	—	—	—	—	—	—	26.01	—	—	—	—	—	—	—
ρ-Cymen-7-ol	—	—	—	—	—	—	—	—	—	—	—	—	28.77	—	—	—	—	—	—	—
*m*-Cymen-8-ol	—	—	—	0	0	—	—	—	—	2.25	—	—	0.54	—	—	0	—	—	0	—
*m*-Cumenol	—	—	—	—	—	—	—	—	—	—	—	—	—	—	—	—	—	—	0.11	—
Davanone	—	—	—	—	—	—	—	—	—	—	—	—	—	—	—	—	—	—	0.42	—
Dihydrocarvone (*cis*)	—	—	—	—	—	—	—	6.85	—	—	—	—	—	—	—	0.64	—	—	—	—
Dihydrocarveol	—	—	—	—	—	—	—	—	—	—	0.81	—	—	—	7.12	—	—	—	—	—
Dihydrocarvone (*trans*)	—	—	—	—	—	—	—	—	—	—	1.09	—	—	—	0	—	—	—	—	0.82
Dill apiole	—	—	—	—	—	—	—	—	—	—	—	—	—	1.56	7.03	—	—	—	—	—
Dihydroactinidiolide	—	—	—	—	—	—	—	—	—	—	—	—	—	6.65	—	—	—	—	—	—
β-Eudesmol	—	—	—	—	—	—	—	—	—	—	—	—	—	—	—	—	—	—	—	0.34
Eugenol	—	22.65	—	—	0.42	—	5.1	—	28.8	—	—	—	—	—	—	42.07	23.43	—	—	0.79
Eugenol acetate	—	0.95	—	—	—	—	—	—	—	—	—	—	—	—	—	—	—	—	—	—
β-Fenchyl alcohol	—	—	—	—	—	—	—	—	—	—	—	—	—	2.2	—	—	—	—	—	—
Fenchone	—	1.21	0	—	—	—	—	—	—	—	—	—	—	—	—	1.51	—	—	—	—
Filifolone	—	—	—	—	—	—	—	—	—	0.79	—	—	—	—	—	0	—	—	—	—
Geranial	—	—	—	—	—	—	—	—	—	—	—	13.72	—	—	—	—	—	—	—	—
Geraniol (*cis*)	—	—	8.82	—	—	—	—	—	—	—	—	—	—	—	—	—	—	—	—	—
Geraniol	—	—	—	—	—	—	2.51	—	2.89	—	—	—	—		—	—	—	—	—	—
Guaiacol	—	—	—	—	—	—	—	—	—	—	—	—	—	0.63	—	—	—	—	—	—
Hepten-2-one, 6-methyl-5	—	0.49	—	—	—	—	—	—	—	—	—	—	—	—	—	—	—	—	—	—
Hexadecanoic acid	7.45	—	—	—	—	—	—	—	—	—	—	—	—	—	—	—	—	—	—	—
α-Humulene	—	—	—	—	—	5.3	—	—	—	—	—	—	—	—	—	—	—	—	—	—
Indole	—	—	5.5	—	—	—	—	—	—	—	—	—	—	—	—	—	—	—	—	—
Intermedeol	—	—	—	—	—	—	—	—	—	—	—	—	—	—	—	—	—	—	0.51	—
Jasmone (*trans*)	—	—	—	—	—	—	—	—	—	—	—	—	—	—	—	—	—	—	—	0.47
Jasmine (*Z*)	—	—	—	—	—	—	—	—	—	—	—	—	—	—	—	—	—	—	0.36	—
Limonene	—	—	—	—	—	—	—	—	2.36	—	—	20.55	—	—	—	—	—	—	—	—
Linalool	—	1.72	36.68	0.86	2.08	0	0	0	0.97	1.68	1.13	—	—	—	—	0.72	—	—	0.79	—
Linalool oxide (*cis*)	—	—	1.24	—	—	—	—	—	—	—	—	—	—	—	—	—	—	—	—	—
Linalool oxide (*trans*)	—	—	0.68	—	—	—	—	—	—	—	—	—	—	—	—	—	—	—	—	—
Menth-2-en-l-ol (*cis*-*p*)	—	—	—	—	—	—	—	—	—	—	—	—	—	—	—	—	—	—	0.48	—
Menthol	—	—	—	—	—	—	—	43.67	—	—	—	—	0	—	—	1.66	—	—		
Menthol (*neo*)	—	—	—	—	—	—	—	7.53	—	—	—	—	—	—	—	—	—	—	—	—
Menthone	—	—	—	—	—	—	—	—	—	—	—	—	—	—	—	1.46	—	—	—	—
ρ-Methylanisole	—	—	—	—	—	—	—	—	—	—	—	—	—	—	—	—	—	—	—	—
Methyl anthranilate	—	—	11.31	—	—	—	—	—	—	—	—	—	7.41	—	—	—	—	—	—	—
Methyl eugenol	—	—	—	—	—	—	2.75	—	0.57	—	—	—	—	0.82	—	—	2.2	—	—	1.92
Methyl hexadecanoate	—	1.43	—	—	0.39	—	—	9.54	—	—	4.63	7.95	1.15	—	—	—	—	5.19	0.39	—
Methyl 5-vinylnicotinate	—	—	—	—	—	—	—	—	—	—	—	—	—	28.37	—	—	—	—	—	—
Methyl jasmonate (*Z*)	—	—	—	—	—	—	—	—	—	—	—	—	—	—	—	—	—	—	0.63	—
Methyl octadecanoate	—	—	—	1.94	—	—	—	0.74	—	—	0.31	—	—	—	—	—	—	—	—	—
Myristcin	—	1.12	—	—	—	—	—	—	—	—	—	—	—	—	—	—	4.54	—	0.59	—
Myrtenol	—	—	—	—	—	—	—	—	—	2.27	—	—	—	—	—	—	—	—	1.27	—
Neral	—	—	—	—	—	—	—	—	—	—	0	6.42	—	—	—	—	—	—	—	—
Nerol	—	2.94	2.74	—	—	—	—	—	—	—	—	3.42	—	—	—	—	—	—	0.32	—
Nerolidol (*trans*)	—	—	0.67	—	—	—	—	—	—	—	—	0.68	—	—	—	—	—	—	—	—
Phenethyl alcohol	—	—	1.54	—	—	0	76.95	0	46.9	0	—	—	—	0.92	—	0	58.78	—	—	—
Pinocarveol (*trans*)	—	—	—	—	—	—	—	—	—	8.02	—	—	—	—	—	—	—	—	—	—
Pinocamphone (*trans*)	—	—	—	—	—	—	—	—	—	0.38	—	—	—	—	—	—	—	—	—	—
Pinocarvone	—	—	—	—	—	—	—	0	0	0.66	—	—	—	—	—	—	—	—	—	—
Piperitenone	—	2.66	—	—	—	—	—	—	—	—	—	—	—	—	—	0.86	—	—	—	—
α-Pinene	—	—	—	—	—	—	—	—	—	—	—	2.78	—	—	—	—	—	—	—	—
Pulegone	—	2.64	—	—	—	—	—	6.46	—	—	3.72	—	—	—	6.12	1.04	—	—	—	—
Sabinenehydrate (*cis*)	—	—	—	—	—	—	—	—	—	—	—	—	0.33	—	—	—	—	—	—	—
Spathulenol	—	—	—	—	—	—	—	—	—	—	—	—	—	—	—	—	—	—	0.92	—
α-Terpineol	—	1.67	29.36	1.09	—	—	—	—	—	1.15	—	6.3	0.74	—	—	0.82	—	—	1.65	—
Terpinene-4-ol	—	—	0.7	—	0.47	—	—	—	—	2.26	—	5.1	4.54	—	—	0.77	—	—	8.15	1.09
α-Terpinenyl acetate	—	—	—	4.81	0	—	—	—	—	—	—	—	—	—	—	—	—	—	—	—
γ-Terpinene	—	—	—	—	—	—	—	—	—	—	—	—	0.35	—	—	—	—	—	—	—
Terpinolene	—	—	—	—	—	—	—	—	—	—	—	—	—	—	—	—	—	—	—	0.61
Thujone (*cis*)	—	—	—	—	—	—	—	—	—	—	—	—	—	—	—	—	—	—	0.92	—
Thujone (*trans*)	—	—	—	—	—	—	—	—	—	—	—	—	—	—	—	—	—	—	6.74	4.36
Thymol	29.01	22.29	—	—	4.41	—	—	6.1	—	32.63	45.19	4.87	—	12.3	7.38	22.49	0.63	9.76	2.79	0.82
Thymol acetate	—	—	—	—	—	—	—	—	—	0.4	—	—	—	—	—	—	—	—	—	—
Verbenol (*trans*)	—	—	—	—	—	—	—	—	—	16	—	—	—	—	—	—	—	—	—	—
Verbenone	—	—	—	—	—	—	—	—	—	5.78	—	—	—	—	—	—	—	—	—	2.63
*o*-Xylene	—	—	—	—	—	—	—	—	—	—	—	—	—	—	1.09	—	—	—	—	—
*p*-Xylene	20.2	—	—	—	—	—	—	—	—	—	—	—	—	2.03	11.64	—	—	—	—	0.47
Yamogi alcohol	—	—	—	—	—	—	—	—	—	—	—	—	—	—	—	—	—	—	—	21.14

**Table 4.** K-Means Cluster Analysis of the Aromatic Waters Constituents.

1	Wormwood, yarrow
2	Azarol hawthorn, basil, frankincense, olive, shadab, valerian, lemon balm
3	Bitter orange, lemon verbena, musk willow
4	Chinese cinnamon
5	Creeping buttercup
6	Cuminum
7	Damask rose, dog-rose, starflower
8	Date palm, oriental plane

According to the hierarchical cluster analysis, azarol hawthorn, basil, frankincense, olive, shadab, valerian, lemon balm, lemon verbena, date palm, bitter orange, and musk willow made a big cluster based on their thymol and carvacrol contents with some subcluster within it. For example, basil, shadab, and lemon balm made a subcluster because of similar thymol (22%-45%) and carvacrol (14%-32%) contents (Figure 1).

Date palm and oriental plane made a subcluster according to K-means, which might be due to the similar content of thymol (6.1%-7.3%), pulegone (6.12%-6.46%), and carvone (7.69%-24.21%) in these aromatic waters.

For many of these aromatic waters, this is the first report on their chemical composition. Since many of these aromatics are said to have multipurpose applications such as cardiovascular, hormonal, neurological, and gastrointestinal effects. In our previous works on hydrosols used for cardiovascular conditions or women’s reproductive and hormonal conditions we have reported chemical composition of some of these aromatic waters such as wormwood, yarrow, oriental plane, and azarol howthorn.^10,11^ On the other hand, it was essential for the current research to find relation between these aromatic waters using cluster analysis. Thus, the previously reported^10,11^ aromatic waters were analyzed again to avoid any variation in results due to the experimental conditions.

There is a good agreement between the results of the current article with the aromatic waters that were reported in the previous works.^10,11^ In most of the cases, the major constituents are the same and the chemical compositions are similar with some degree of variation in constituents’ percentages. For some other aromatic waters, such as shadab, lemon verbena, cuminum, Chinese cinnamon, bitter orange, and basil, this is the first report on chemical constituents of their hydrosols thus, it was not possible to compare the results of the current research with others but the major components of the reported essential oils are summarized in Table 5. Considerable differences can be observed by comparing aromatic waters and reported essential oils for these plants. For cuminum, the major components in the aromatic water are *trans*-caryophyllene, 3,4-dimethoxytoluene and caryophyllene oxide while main compounds in cuminum essential oil are cuminal and cuminic alcohol (Table 5). In case of lemon balm and frankincense, carvacrol, thymol, and linalool constituted the main part of aromatic water compositions (Table 3) while the major components of the essential oil of these plants (Table 5) are citronellol, δ-3-carene, and in some cases, carvacrol with citronellal and geraniol.^12^ Significant diffrences can also be found between compositions of damask rose, bitter orange, dog-rose, valerian, and musk willow aromatic waters and essential oils (Tables 3 and 5). These diffrence between aromatic water and essential oil compositions may arise from polarity and solubility of volatile compositions in water.^10,11^ It seems that due to different chemical composition, it is essential to consider different biological activities for aromatic waters compared with pure essential oils.

**Table 5.** Profile of Essential Oils Reported in Literature for the Plants Being Used to Prepare Aromatic Waters for Mental Health and Neurological Conditions.

Plant Name	Profile of Essential Oils Monoherbal Aromatic Waters	References
Azarol howthorn	Viridiflorol, borneol, eicosane, heneicosane, tricosane, squalene, (*E*)-2-hexenal, butyl butyrate, linalool, butyl hexanoate, methyl octanoate, pentyl hexanoate, and hexyl hexanoate	13, 14
Basil	Estragole, linalool, methyl cinnamate, α-cadinol, eugenol, 1,8-cineole, methyl eugenol, α-bergamotene	15
Bitter orange	*trans*-β-Bergamotene, β-santalene, germacrene-B and β-sesquiphellandrene, hexanol, α-terpinene, *cis*-β-ocimene, *cis*-sabinene	16, 17
Chinese cinnamon	3-Methoxy-1,2-propanediol, *trans*-cinnamaldehyde, *o*-methoxy-cinnamaldehyde, eugenol, coumarin	18
Creeping buttercup	Methyl linoleate, carvacrol methyl ether, globulol, aromadendrene, phytol, α-farnesene, α-terpinyl acetate, β-ocimene, and fatty acid derivative	19
Cuminum	Cuminal, cuminic alcohol, γ-terpinene, *p*-cymene, β-pinene	20
Damask rose	Citronellol, nerol, geraniol, nonadecane, 2-phenylethyl alcohol, geranyl acetate	21
Date palm	(*E*)-β-ionone, (*E*)-2-tridecene, limonene, (*E*)-geranylacetone, decanal, ethyl decanoate, ethyl acetate, 2-propanol, isoamyl alcohol	22, 23
Dog-rose	Vitispirane, α-dehydro-*ar*-himachalene, spathulenol, β-caryophyllene oxide	24
Felty germander	α-Pinene, β-pinene, p-cymene, β- caryophyllene, pinocarveol, spathulenol, eudesmol, cadinol	25, 26
Frankincense	α-Pinene, camphene, verbenene, β-pinene, myrcene, limonene	27, 28
Lemon balm	*trans*-Carveol, citronellol, δ-3-carene, citronellal, geraniol, 1-octene-3-ol and spathulenol	29
Lemon verbena	l,8-Cineole, geranial, 6-methyl-5-hepten-2-one, neral, limonene, β-caryophyllene, *ar*-curcumene, spathulenol	30, 31
Musk willow	1,4-Dimethoxybenzene, phenylethyl alcohol, carvone, metheleugenol, citronellol, 4′-methoxyacetophenone	32
Olive leaves	(*E*)-2-hexenal, (*E*,*E*)-R-farnesene, linalool, α-caryophyllene, valencene, 4-terpineol, (*E*)-ocimene, *p*-cymen-8-ol, carvone, R-humulene, germacrene D, *trans*-nerolidol	33
Starflower	α-Cadinene, viridiflorol, α-muurolene, ledene, α-calacorene, α-cadinene	34
Valerian	Camphene, α-campholene aldehyde, bornyl acetate, α-gurjunene, α-cedrane, epizonaren, germacrene-B, valerenal	35, 36
Wormwood	(*Z*)-epoxyocimene, chrysanthenyl acetate, β-thujone, *trans*-sabinyl acetate, sabinene	37, 38
Yarrow	Chamazulene, 1,8-cineole, α-pinene, β-pinene, thujane, *p*-menthane, piperitone, linalool, β-caryophyllene, borneol, camphor, nerolidol, and limonene	39

## Conclusion

The present investigation introduced some aromatic waters that are used in Persian nutrition culture and folk medicine for nurological conditions and maintaining mental health. Based on this reserch chemical compositions of these aromatic waters are remarkably different from the essential oils of the plants used to prepare them. These plants originated from a vriety of genus and families but using cluster analysis (hierarchical cluster analysis and K-means) showed that some similarity can be identified between their chemical compositions. Thymol, phenethyl alcohol, carvacrol, eugenol, and/or camphor were the major constituents in most of the aromatic waters. This study was not designed to evaluate the efficacy of these aromatic waters (hydrosols), but centuries of production and consumption of these aromatic waters in Persian folk medicine and nutrition culture might be related to their efficacy. This research may present a valuable line for developing functinal beverages for mental health or neurological conditions. Also, scientific evaluation of these aromatic waters constituents may lead to some new therapeutic agents.
